# Efficient and Accurate Synapse Detection With Selective Structured Illumination Microscopy on the Putative Regions of Interest of Ultrathin Serial Sections

**DOI:** 10.3389/fnana.2021.759816

**Published:** 2021-11-15

**Authors:** Gyeong Tae Kim, Sangkyu Bahn, Nari Kim, Joon Ho Choi, Jinseop S. Kim, Jong-Cheol Rah

**Affiliations:** ^1^Korea Brain Research Institute, Daegu, South Korea; ^2^Department of Biomedical Engineering, Ulsan National Institute of Science and Technology, Ulsan, South Korea; ^3^Department of Biological Sciences, Sungkyunkwan University, Suwon, South Korea; ^4^Department of Brain and Cognitive Sciences, Daegu Gyeongbuk Institute of Science and Technology, Daegu, South Korea

**Keywords:** structured illumination microscopy, array tomography, synapse location, posterior medial nucleus, barrel cortex

## Abstract

Critical determinants of synaptic functions include subcellular locations, input sources, and specific molecular characteristics. However, there is not yet a reliable and efficient method that can detect synapses. Electron microscopy is a gold-standard method to detect synapses due to its exceedingly high spatial resolution. However, it requires laborious and time-consuming sample preparation and lengthy imaging time with limited labeling methods. Recent advances in various fluorescence microscopy methods have highlighted fluorescence microscopy as a substitute for electron microscopy in reliable synapse detection in a large volume of neural circuits. In particular, array tomography has been verified as a useful tool for neural circuit reconstruction. To further improve array tomography, we developed a novel imaging method, called “structured illumination microscopy on the putative region of interest on ultrathin sections”, which enables efficient and accurate detection of synapses-of-interest. Briefly, based on low-magnification conventional fluorescence microscopy images, synapse candidacy was determined. Subsequently, the coordinates of the regions with candidate synapses were imaged using super-resolution structured illumination microscopy. Using this system, synapses from the high-order thalamic nucleus, the posterior medial nucleus in the barrel cortex were rapidly and accurately imaged.

## Introduction

Despite the importance of synapse mapping, an appropriate method that can expeditiously and reliably map the location of synaptic inputs is currently lacking. Developing such a method is difficult because synapses are often smaller than the light diffraction limit and densely packed in a three-dimensional volume (Schüz and Palm, 1989; DeFelipe, 1999). Thus, conventional fluorescence microscopy (FM) does not provide a sufficient resolution (Mishchenko, 2010). Electron microscopy (EM) provides a sufficient resolution to trace neuronal processes and detect synapses, but it remains challenging to reconstruct a volume that is large enough to cover a meaningful fraction of the neural circuit (Lichtman and Denk, 2011; Briggman and Bock, 2012; Kim et al., 2012; Zheng et al., 2018). Furthermore, compatible synaptic input labeling methods are critically limited in EM (however, see Li et al., 2010; Atasoy et al., 2014; Zhang et al., 2019) and often have compromised preservation of ultrastructure (Li et al., 2010; Lam et al., 2014).

Various light microscopic imaging techniques have been developed for the sake of circuit reconstruction, each with its pros and cons (Kim et al., 2012; Wickersham and Feinberg, 2012; Schoonover et al., 2014; Chen et al., 2015). One promising method with which to enhance synapse detection accuracy using conventional FM is array tomography (AT), which is an imaging technique based on iterative FM on a serially sectioned volume of tissue followed by computational reconstruction (Micheva and Smith, 2007). Given that the axial resolution is determined by tissue thickness, isotropic or better axial resolution can be achieved relatively easily. Theoretical and experimental studies have demonstrated that, using AT, synapses can be detected with high accuracy and reliability (Micheva and Smith, 2007; Rah et al., 2013; Bloss et al., 2016).

Nevertheless, AT has not been extensively adopted in neuroscience (Oberti et al., 2011; Rah et al., 2013; Bloss et al., 2018). The limited applications of AT in neuroscience could be due to the relatively long imaging time compared with other LM methods (Feinberg et al., 2008; Kim et al., 2012) and the relatively low accuracy of synapse detection compared with EM (Rah et al., 2013; Bloss et al., 2016).

Structured illumination microscopy (SIM) is a super-resolution microscopy that improves lateral resolution by a factor of two or more, thus providing a resolution of 100 nm or higher. The super-resolution images are calculated from the Moiré fringes generated as the product of unknown biological structure and the known spatially patterned illumination (Gustafsson, 2000; Gustafsson et al., 2008). Given that Moiré fringes tend to be coarser and within the range of diffraction limit, normally inaccessible high-resolution information in Fourier space of the biological structure can be extracted (Gustafsson et al., 2008). On the other hand, one theoretical estimation predicted that isotropic 100 nm resolution microscopy with enough fluorophores can guarantee accurate synapse detection (Mishchenko, 2010). For that reason, SIM in combination with AT is an attractive candidate method.

To enable rapid neuronal reconstruction with precise synapse identification, we developed an imaging technique called structured illumination microscopy on the putative region of interest on ultrathin sections (SIM-PRIUS). Briefly, we combined two sets of images, including low-magnification images of dendritic structure with 20x FM, and selective SIM images of regions with putative synapses-of-interest. Using this technique, we efficiently measured the number of synapses originating from the high-order thalamic nucleus, the posterior medial nucleus (POm), on layer (L)5 pyramidal neurons of the barrel cortex.

## Materials and Methods

### Overall Procedure

**FIGURE 1:**
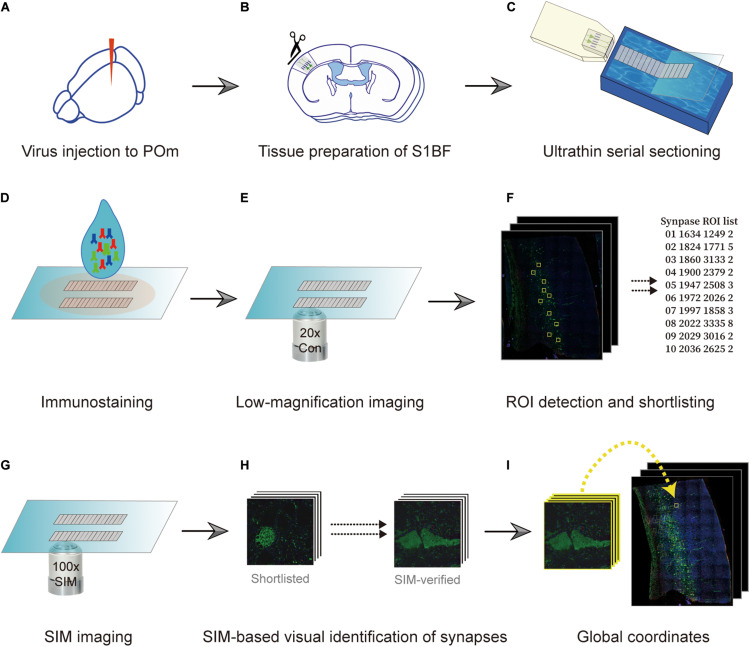
Overview of the imaging technique. **(A)** Fluorescence labeling of synapses-of-interest. Neurons in the posteromedial nucleus of the thalamus (POm) were labeled using stereotactically delivered adeno-associated virus (serotype 1) transduction of tdTomato in a Thy1-EGFP (type M) mouse. After 4 weeks, the mouse was sacrificed and fixed with 4% paraformaldehyde. **(B)** Embedding. Subsequently, the S1BF region was embedded in Lowicryl resin. **(C,D)** Serial sectioning and immunostaining. A series of 90 nm sections were collected **(C)** and immunostained with antibodies against synaptophysin-1, GFP, and tdTomato **(D)**. **(E,F)** Low-magnification imaging and ROI detection. The series of sections were imaged at 20x resolution, and the global coordinates of ROIs were computed based on the existence of overlapping color channels. **(G)** SIM imaging. ROIs were revisited, and SIM images of the regions were acquired. **(H,I)** Determination of synapse locations. SIM images were post-processed to render the synapse images and visually determine if the structure indeed formed synapses **(H)**. True synapses were relocated on the low-magnification image with image registration **(I)**.

Specimens used in the procedure were prepared in the same way as those prepared for AT (Micheva and Smith, 2007). Briefly, pre- and postsynaptic neurons were fluorescently labeled using an adeno-associated virus (AAV) and transgenic mouse (Figure 1A). We exemplified the POm inputs on L5 pyramidal neurons in the barrel field of the primary somatosensory cortex (S1BF). POm neurons were densely labeled with AAV to avoid the bias caused by the uneven labeling of presynaptic neurons. Therefore, spillover labeling of neurons in neighboring thalamic nuclei, such as the ventral posteriormedial nucleus, was inevitable. For convenience, we called the example synapses with axons from the tdTomato-expressing neurons enriched in POm as POm synapses. We took advantage of the transgenic mouse line whose L5 pyramidal neurons are sparsely labeled with an enhanced green fluorescent protein (EGFP) in S1BF. After approximately 4 weeks, the S1BF area was embedded in the Lowicryl HM20 and serially sectioned into 90 nm sections using an ultramicrotome (Figures 1B,C). The serial sections were immunostained with synaptophysin 1, GFP, and tdTomato antibodies to label synapses and to boost the genetically encoded fluorescent signals that may have been diminished during the embedding process (Figure 1D). We first acquired images of the whole serial sections at a low magnification (20x) to acquire structural information and define locations of interest (Figure 1E). The regions of interest (ROIs) vary according to the purpose of a study; we set the ROIs as the POm synapses on L5 pyramidal neurons in S1BF (Figure 1F). Candidate synapses were selected based on the adjacency of the axons and dendrites of interest, assuming that synapses-of-interest cannot be present without structural adjacency. We then revisited the recorded regions of synapse candidates and re-imaged them using SIM to determine whether the structural adjacency forms synapses (Figure 1G). To enhance the accuracy in determining synapses, we acquired three serial images of the ROI by imaging the exact relative locations of the preceding and the following sections (Figure 1H). The global coordinates of candidates verified as synapses were noted to calculate synapse density (Figure 1I).

### Animal

All animal experiments followed the procedures approved by the Korea Brain Research Institute (KBRI). A 10-week-old Tg(Thy1-EGFP)MJrs (Feng et al., 2000) female mouse (Jackson Laboratory, ME, United States) was used in the current study.

### Stereotaxic Surgery

The synaptic inputs from the POm were labeled by stereotaxic delivery of AAV transduction of tdTomato in the Thy1-EGFP mouse (Figure 1A; Feng et al., 2000). The mouse was anesthetized with 2% isoflurane and secured in stereotaxic apparatus (Kopf Instruments, CA, United States). The response to toe pinches was examined to monitor the depth of anesthesia. The flow and concentration of isoflurane were adjusted as needed. For labeling of POm inputs in S1BF, 50 nl of AAV1-CAG-tdTomato-WPRE-SV40 (Addgene, MA, United States) was delivered to the POm (dorsal-ventral: −2.9 mm, medial-lateral: +1.25 mm, anterior-posterior: −2.06 mm). Four weeks after the injection, the mouse was deeply anesthetized using intraperitoneally injected pentobarbital sodium (50 mg/kg body weight) and underwent transcardial perfusion of fixative, 4% paraformaldehyde (Electron Microscopy Sciences, PA, United States) in Tris-buffered saline (VWR Life Science, PA, United States). After further incubation in fixatives overnight, the extracted brain was rinsed and coronally sectioned at 300 μm using a vibratome (Leica biosystems, IL, United States; Figure 1B). The location of the injection site was examined with reference to the mouse brain atlas (Franklin and Paxinos, 2008) using QuickNII (Puchades et al., 2019).

### Tissue Embedding and Serial Sectioning

S1BF with proper labeling was dissected and embedded in HM20 (Electron Microscopy Sciences, PA, United States) using a low-temperature embedding system (Leica Biosystems, IL, United States). The embedded tissue had a block face size of 672 μm × 1,088 μm, and was serially sectioned at 90 nm using an ultramicrotome (RMC Boeckeler, AZ, United States; Figure 1C). Thirty-five consecutive sections were collected on coverslips (Electron Microscopy Sciences, PA, United States) for subsequent immunostaining and imaging (Figure 1E).

### Immunostaining

Sections were incubated with 50 mM glycine (Sigma, MO, United States) at room temperature for 5 min to remove free aldehyde groups. The sections were permeabilized with 0.05% Triton X-100 (Fisher Scientific, NH, United States), and incubated with blocking solution (0.1% normal donkey serum (Millipore, MA, United States) and 0.05% Tween20 (Sigma, MO, United States) in Tris-buffered saline for 10 min at room temperature. The sections were then incubated with the following primary antibodies overnight at 4°C: anti-GFP antibody 1:500 (Abcam, Cambridge, United Kingdom), anti-synaptophysin-1 1:250 (Synaptic System, Göttingen, Germany), and anti-Living Colors DsRed 1:100 (Takara Bio, CA, United States) diluted in the blocking solution. After washing away the unbound primary antibodies, sections were incubated with the following fluorophore-conjugated secondary antibodies for approximately 1.5 h at room temperature: Alexa Fluor 488 AffiniPure Donkey Anti-Chicken 1:1000 (Jackson ImmunoResearch, PA, United States), Alexa Fluor 594 AffiniPure Donkey Anti-Rabbit 1:100 (Jackson ImmunoResearch, PA, United States), and Alexa Fluor 647 AffiniPure Donkey Anti-guinea pig 1:250 (Jackson ImmunoResearch, PA, United States) diluted in the blocking solution. Then, the sections were mounted on microscope slides with 200 μl ProLong Diamond Antifade Mountant (Invitrogen, MA, United States) and covered with CoverWell incubation chamber gaskets (Life Technologies, CA, United States).

### Conventional Fluorescence Imaging and Alignment

**FIGURE 2:**
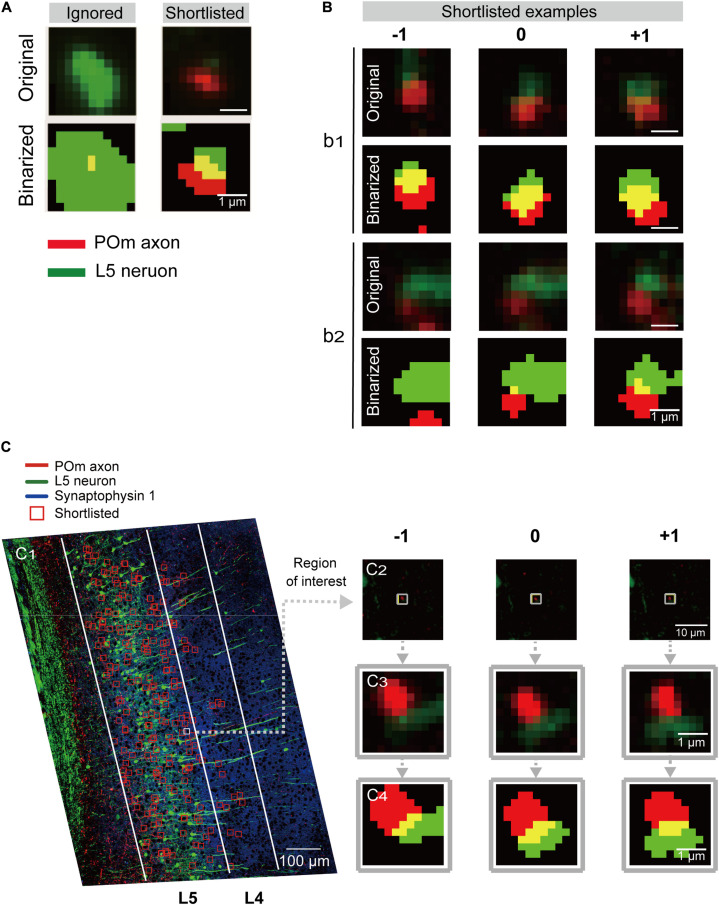
Detection and shortlisting of synapse candidates. **(A)** Examples of the ROIs detected by overlapping of the binarized low-magnification images from two channels (see section “Materials and Methods” for details). ROIs without a continuous structure in the neighboring sections or ones that sat in the middle of large structures were ignored (left). Representative example of the shortlisted ROIs (right). Red and green signals were from POm axons and L5 pyramidal neurons, respectively. The overlapping region is shown in yellow. **(B)** Examples of shortlisted ROIs and the same coordinates of their preceding(–1) and following (+1) section. Scale bar = 1 μm. Pixel intensity plots along the red and green lines shown in the inset images in **(C)**. The pixel intensities were shown along the horizontal (green) and vertical (red) lines in the conventional (dotted) and SIM (solid) images. **(C_1_)** Examples of shortlisted ROIs overlaid onto the z-projected low-magnification image. The two target areas, L4 and L5, are outlined with white lines. Scale bar = 100 μm. **(C_2_)** An example of the shortlisted ROIs (middle) and the same coordinate of the preceding (left, −1) and following (right, +1) section. Scale bar = 1 μm. **(C_3_)** Magnified images of **(C_2_)**. **(C_4_)** The binarized and dilated images of **(C_3_)**. Scale bar = 1 μm.

The low-magnification imaging was performed on the same microscope with the subsequent SIM imaging (Nikon, Tokyo, Japan), but with a 20x objective lens and halogen illuminator. 20x images covering the entire sections were acquired using a multi-tile imaging macro (Supplementary Data 1) of the image acquisition software, NIS-Elements (Nikon, Tokyo, Japan; Figure 2). To minimize imaging time, we detected regions within which axons and dendrites of interest were in proximity because synapses-of-interest cannot exist without the axons and dendrites. For selective and reliable detection of the physical contacts, we binarized the images of each channel. Specifically, 9 × 10 tiles of 20x images were stitched using the “Stitch Grid of Images” (Preibisch et al., 2009) and aligned between layers using the Thin Plate Spline Transform-based elastic layer alignment in TrakEM2 (Bogovic et al., 2016).

### Regions of Interest Detection

Out of the images covering the entire sections, we set the target detection area, within which ROIs were searched (Figure 1F). To exemplify, we set L4 and L5 as the two target areas to detect candidates of POm synapses in the current study (Figure 2C white lines). To cancel the brightness variances between image tiles, the mean and variance of the brightness of each slice image were normalized using the following formula (Tortoli and Masotti, 1996):


I⁢m⁢g/s⁢t⁢d⁢(I⁢m⁢g)*σ-m⁢e⁢a⁢n⁢(I⁢m⁢g/s⁢t⁢d⁢(I⁢m⁢g)*σ)+μ

We then homogenized the background brightness within the tiled image by subtracting the local brightness tendency calculated by Gaussian blurring of the image. Consequently, three channels were binarized using Renyi’s entropy method (Sahoo and Arora, 2004). Each positive pixel was dilated to 9 pixels to minimize false-negative detection error (8% ± 3.5 (mean ± standard deviation) resulting from the low magnification. When the overlapped signal was detected, a bounding box with the size of SIM’s field of view, centering at the overlapping pixel, was generated to define the coordinate of subsequent SIM imaging. To minimize the redundant SIM imaging resulting from the overlap of bounding boxes, we merged all the bounding boxes and generated a new set of bounding boxes that covered all the areas with minimal overlap.

### Regions of Interest Shortlisting

ROIs in the target area were shortlisted according to several characteristics of the synapses-of-interest (Figure 1F). Namely, POm synapses on L5 pyramidal neurons should have red fluorescence from tdTomato expression in POm neurons in proximity to EGFP-expressing dendritic spines. Also, because target structures are a part of continuous neuronal processes, the red-green signal should reappear at the exact or adjacent coordinates of neighboring section(s). Therefore, ROIs detected in the middle of a large structure (Figure 2A) or ROIs without continuity at the adjacent sections were regarded as noise and removed. Moreover, the continuous synapse signals of adjacent sections were bound together to a set of synaptic image series by adding the same coordinates of the one or two preceding and following sections into the ROI list (Figure 2). To acquire the global coordinates on the slide of each ROI, the coordinates within the target areas were first translated to the global coordinates, and the inverse function of the transformation was then applied.

### Structured Illumination Microscopy Imaging of the Shortlisted Regions of Interests

**FIGURE 3:**
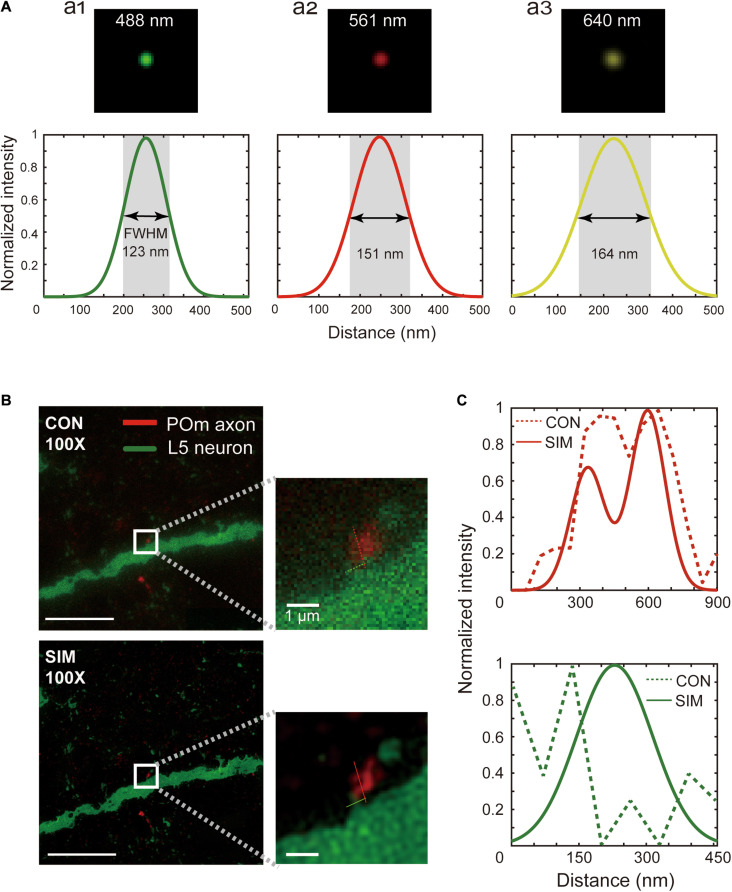
Spatial resolution of SIM images. **(A)** Point spread function measured with the excitation wavelengths used for SIM imaging, 488 nm (a_1_), 561 nm (a_2_), and 640 nm (a_3_). The full width half maximum of a fluorescent bead was measured at these wavelengths. **(B)** A relatively small pre- and postsynaptic structure image acquired by conventional microscopy (upper) and SIM (below), and a zoomed-in view (white box, inset). Scale bars represent 10 μm (left) and 1 μm (inset). **(C)** Pixel intensity plots along the red and green lines shown in the magnified images of conventional (dotted) and SIM (solid) images.

Commercial SIM equipment (Nikon, NSIM) with a 100x oil-immersion objective lens (Nikon, SR Apo TIRF 100x, N.A.1.49) and electron-multiplying charge-coupled device (Andor, DU-897E) was used with an acquisition speed of 50 frames/second. All optics were controlled using NIS-Elements (Nikon, Japan). We verified the resolution of the microscope with 0.1 μm diameter fluorescent beads emitting the three imaging wavelengths of 488 nm, 561 nm, and 640 nm (Thermo fisher Scientific, Tetraspek microsphere; Figure 3). SIM images were acquired for all ROI coordinates using a novel macro script (Supplementary Data 1) that adjusts imaging parameters and electric devices at each coordinate of the bounding box (Figure 1G and Supplementary Data 1). For every new ROI, the laser power was adjusted according to the intensity profile of the location to set the intensity profile that would be adequate for SIM imaging. When the intensity profile could not be adjusted sufficiently using laser power alone, exposure or electron-multiplying gain of the camera was adjusted. However, this function was scarcely used. SIM images were acquired. Five set images were acquired with three migrating high-frequency sinusoidal stripes at 120° to each other. Upon completing the raw image acquisition, the sample stage moved to the subsequent ROI on the list. This process was repeated iteratively to image all the shortlisted ROIs. Grating directions and amplitude were adjusted for each excitation wavelength.

## Results

### Structured Illumination Microscopy Provided Better Accuracy on Physical Contacts Than Conventional Fluorescence Microscopy

We first ensured that the SIM microscope met the expected resolution by measuring the point spread function using 100 nm fluorescent beads with the three excitation wavelengths used for synapse detection (Figure 3A, upper). The full widths at half maximum with 488 nm, 561 nm, and 640 nm excitation wavelengths are shown in Figure 3A (lower). Indeed, the full width at half maximum was in the range of the theoretical expectation (123 ± 7.18 nm, 151 ± 7.90 nm, and 164 ± 8.33 nm at the 488 nm, 561 nm, and 640 nm excitation wavelengths, mean ± standard deviation).

One theoretical prediction has suggested that isotropic 100 nm resolution is sufficient for accurate synapse detection (Mishchenko, 2010). Indeed, ambiguous physical contacts of pre- and postsynaptic structures by the conventional microscope (Figure 3B, upper, and Figure 3C, upper) were readily resolved as clear physical contacts using SIM with a 90 nm axial resolution (Figure 3B, lower, and Figure 3C, lower). Given that the false-negative synapse detection rate of SIM-PRIUS is critically dependent upon the reliable detection of the physical contacts at 20x, we further examined how well physical connections observed with SIM can be detected at 20x. This revealed that ROI bounds detected in 20x covered approximately 93% ± 3.5 (mean ± standard deviation) of the intersections found in SIM, which suggests that missed synapses by SIM-PRIUS are negligible (Supplementary Figure 2). Furthermore, the physical connections apparent using the conventional microscope (Supplementary Figure 1A, upper, and Supplementary Figure 1B) were identified as bypassing structures with gaps of around 160 nm (Supplementary Figure 1A, lower, and Supplementary Figure 1C).

Therefore, the circuit reconstruction using SIM on the serial sections provided a better synapse detection accuracy than conventional AT. However, imaging one tile using SIM took approximately 3 minutes, which highlights the need for better efficiency.

### Detection of the Synapses-of-Interest

**FIGURE 4:**
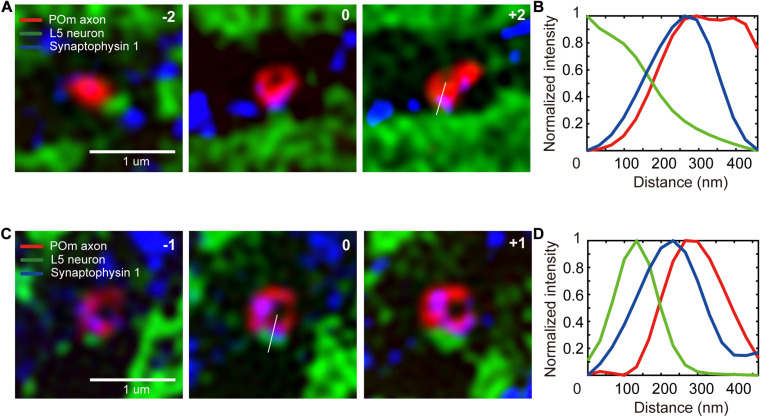
Detection of the synapses-of-interest. **(A,C)** A series of SIM images of synapses-of-interest (scale bar = 1 μm). The numbers at the top right-hand corner indicate the relative z location from the section in which a synapse was identified. **(B,D)** Signal intensity histogram of the three channels across the white lines shown in the image.

We selected the ROI as the region within which axons and dendrites of interest intersect. To compare the numbers of POm synapses on a unit area of dendrites in L4 against L5, we set the two layers as target areas. A total of 3,268 SIM images were obtained on the candidate locations in the two target areas, including 3–5 serial images of the shortlisted ROIs (Figures 4A,C). A total of 1,003 putative synapse image series were visualized using our novel graphical user interface (Supplementary Data 1 and Supplementary Figure 3), and synapses were visually identified (Figures 1H, 4). We considered axons of the true synapses-of-interest to be those with a red signal from tdTomato expressed in the POm axons and a blue signal from immunostaining of synaptophysin 1. As previously noted, the intensity of the synaptophysin 1 signal tends to increase gradually toward green at the synaptic structure (Rah et al., 2013; Figure 4).

### Synapses-of-Interest in L4 and L5

**FIGURE 5:**
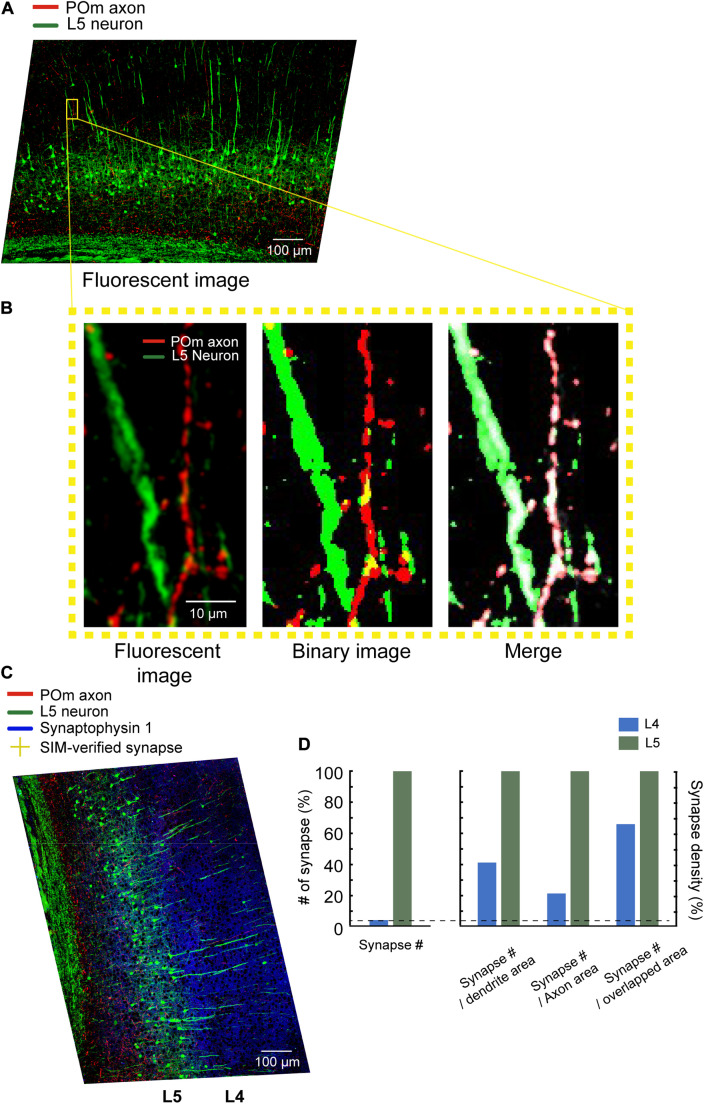
Image segmentation and quantitative analysis. **(A)** Maximum intensity projected 20x images. Scale bar = 100 μm. **(B)** Left: a zoomed-in image of the area indicated with a yellow box. Middle: The binarized image of the same area. Right: The overlaid original images (both channels are shown in white) with segmented axons (red) and dendrites (green). Scale bar = 10 μm. **(C)** Relocated POm synapses confirmed by SIM images (yellow crosses) on the z-projected 20x image. Cortical layers were defined by the density of cell bodies, as shown by white lines. Scale bar = 100 μm. **(D)** The number of POm synapses on the L5 pyramidal neurons in L4 and L5 (left). The number of POm synapses in the unit area of axons, dendrites, and the intersections (right).

We compared the number of POm synapses in L4 and L5. The POm synapses were observed 26 times more frequently in L5 than in L4 (17 and 447 in L4 and L5, respectively; Figure 5D). We then examined whether the areas of dendrites, axons, or contacts may explain the differences in the numbers of POm synapses. Although the dendrites of L5 pyramidal neurons occupied an appreciably greater area in L5 than in L4, the areas in L5 were only about 10-fold greater than in L4 (7,777.5 μm^2^ in L4 and 83,857 μm^2^ in L5; Figures 5A,B). While POm axons are densely bifurcated in L5, as shown previously (Deschênes et al., 1998; Meyer et al., 2010), the density of axons in L5 was only about 5.5-fold greater (2,497.6 and 13,813 μm^2^ in L4 and L5, respectively); this suggests that the density of neither dendrites nor axons can fully account for POm synapse density of the two layers. Finally, we investigated whether areas of intersections between axons and dendrites can proportionally predict the differences in the number of POm synapses. POm axons and dendrites overlapped in around a 17.3-fold greater area in L5 than in L4 (279 μm^2^ in L4, 4830 μm^2^ in L5; Figure 5D), which suggests that the intersection areas can predict the ratio of POm synapses better than areas of dendrites or axons, but not accurately (Figure 5).

## Discussion

Arguably, the greatest challenge in circuit reconstruction is that the nm-scale synaptic connectivity must be reliably and selectively found within a relatively large microcircuit that is often on the mm-scale or larger. To overcome this problem, several studies have achieved ground-breaking advances in large-scale EM image acquisition (Eberle et al., 2015; Morgan et al., 2016; Zheng et al., 2018; Shapson-coe et al., 2021) and analysis (Januszewski et al., 2018; Li et al., 2019). Despite the efforts to improve EM-based neural circuit reconstruction, the lack of an efficient labeling method has prevented this method from being universally used for morphological circuit analysis. Although ingenious methods of genetic labeling have been developed (Li et al., 2010; Atasoy et al., 2014; Zhang et al., 2019), pretreatment of biological specimens is often accompanied by suboptimal ultrastructure preservation. AT-based neural circuit reconstruction is the method of choice in many studies because of the niche (Oberti et al., 2011; Rah et al., 2013; Smith, 2018; Simhal et al., 2019; Kim et al., 2021). These studies have recognized the advantages of AT, such as molecular and genetic labeling of synapses, which enables the identification of molecular profiles and synapse origins. Nonetheless, AT has its own shortcomings, including limited synapse detection accuracy, a false-positive rate of around 20% and a false-negative rate of approximately 14%, and long imaging time due to an inefficient imaging strategy. With SIM-PRIUS, we overcame two of these significant shortcomings, namely, the accuracy and imaging burden. SIM-PRIUS provided a significantly improved lateral (123 ± 7.18 nm) and axial (90 nm) resolution. It was evident that synapses can be identified with a lower false-positive (Figure 3 and Supplementary Figure 1) detection rate, as predicted and reported previously (Mishchenko, 2010; Liu et al., 2018; Crosby et al., 2019). Moreover, depending on the resolution required for any given research question, one could further improve the resolution by using non-linear SIM with thinner sectioning (Rego et al., 2012). Therefore, SIM-PRIUS can be applied broadly.

While it is obvious that SIM-PRIUS provides a lower false-positive detection rate than conventional AT (Figure 3), the risk for false-negative detection is greater than in conventional AT. However, we found that SIM-PRIUS missed less than 8% ± 3.5 (mean ± standard deviation) of putative synapses (Supplementary Figure 2).

We used a simple pixel-based ROI categorization to pre-select the physical connections in the current study. However, using a machine learning-based algorithm with the aid of SIM-validated synapse images could enhance the prediction accuracy and further reduce imaging time and false-negative detection. Typically, excitatory synapses are formed on small structures as dendritic spines, and synaptic vesicles are denser near the synaptic contacts (Rah et al., 2013; Figure 4). The identification of such features is difficult using pixel-based categorization, but can be reflected using a machine learning algorithm (Kreshuk et al., 2011; Schubert et al., 2019).

The imaging burden could also be reduced significantly. We found that when the synaptic inputs originated from the POm on L5 pyramidal neurons, only the putative physical contacts between POm axons and L5 pyramidal neurons can be pre-selected and re-imaged with SIM, rather than imaging the entire cuboid (Figure 2). Considering the imaging field of view of SIM, 40 × 44 image tiles were required to cover the target 1.3 × 1.45 mm^2^ area. In other words, given that approximately 3 min were required to complete a single tile SIM image, 3,080 h of imaging was anticipated to reconstruct the target volume (3 min × 40 × 44 tiles × 35 sections). Instead, using SIM-PRIUS, the acquisition of the target area was completed in 163.4 h. This meant that 3,268 pre-selected areas were imaged using SIM in SIM-PRIUS, rather than 61,600 images. Thus, the required imaging time was reduced by 95% by using SIM-PRIUS compared with reconstructing the entire volume using SIM.

To verify the practicality of SIM-PRIUS, we assessed the number of POm synapses in L4 and L5 in dendritic areas (Figure 5). Using stereological analysis of correlative EM, DeFelipe and Fairen (1993) have estimated the number of symmetric and asymmetric synapses in a unit area of neuropil in each layer of the barrel cortex, where ∼50 synapses were observed in 10 × 10 μm^2^ neuropil of L5 in the adult mouse barrel cortex. We found ∼0.5 POm synapses/10 × 10 μm^2^ of postsynaptic neurons (Figure 5D). Direct comparison of the density is not feasible given that DeFelipe assessed density in the area of neuropil, avoiding soma and large dendrites (DeFelipe and Fairen, 1993), whereas we measured the entire area of postsynaptic neurons without including all the axons in the area. Recent volumetric reconstruction of the human cerebral cortex assessed the volumetric contribution of different fractions of cells (Shapson-coe et al., 2021). According to this work, the area of unmyelinated axons and dendrites is 1.6-fold greater than the area of dendrites and soma, which would lead us to expect 0.8 (0.5 × 1.6) POm synapses in the same area, that is, around 1.6% of the synapses in L5. Using SIM-PRIUS, we found that the number and density of POm synapses are drastically lower in L4 than those in L5. This finding corroborates previous observations of the distribution of POm synapses and axonal bifurcation in S1BF (Petreanu et al., 2009; El-Boustani et al., 2020). It should be noted that the estimation is rough and based on uncertain assumptions; namely, the areal fractionation is from a different cortical area of a different species. The number and distribution of synapses cannot be conclusive without repetitive experiments, especially given the technical challenges of selective and saturated labeling of the POm nucleus. We labeled POm in saturation in the current study, which unavoidably leads to spillover labeling of neurons in the ventroposterior medial nucleus, as evident in POm synapses in L4 and L5B (Figure 5C).

In the current study, we developed an imaging technique, SIM-PRIUS, to enable efficient and accurate detection of synapses-of-interest in a large area of neural circuits. This method can be applied by a wide range of studies to examine sparse and small candidate structures in a large volume of tissue.

## Data Availability Statement

The raw data supporting the conclusions of this article will be made available by the authors, without undue reservation.

## Ethics Statement

The animal study was reviewed and approved by Institutional animal care and use committee of the Korea Brain Research Institute.

## Author Contributions

J-CR designed the experiment with input from JC and JK as well as the other authors. GTK and NK collected the data. GTK, NK, SB, and JC analyzed the data with the supervision of JK and J-CR. GTK, NK, and J-CR interpreted the data and wrote the manuscript with input from the other authors. All authors contributed to the article and approved the submitted version.

## Conflict of Interest

The authors declare that the research was conducted in the absence of any commercial or financial relationships that could be construed as a potential conflict of interest.

## Publisher’s Note

All claims expressed in this article are solely those of the authors and do not necessarily represent those of their affiliated organizations, or those of the publisher, the editors and the reviewers. Any product that may be evaluated in this article, or claim that may be made by its manufacturer, is not guaranteed or endorsed by the publisher.

## Supplementary Material

Supplementary Figure 1 Spatial resolution of SIM images. **(A)** A relatively small pre- and postsynaptic structure image acquired by conventional microscopy (upper) and SIM (lower), and a zoomed-in view (white box, inset). Scale bars represent 10 μm (left) and 1 μm (inset). **(B,C)** Pixel intensity plots in the magnified images of conventional (dotted) and SIM (solid) images.

Supplementary Figure 2 Hit and false-negative synapse detection rate of SIM-PRIUS. Comparing of 20x conventional image and 100x SIM images on the same area and quantifying how well the physical connections observed with SIM can be detected in 20x images.

Supplementary Figure 3 Graphical user interface (GUI) for synapse validation. SIM image of the ROI can be visualized with custom-built graphical user interface (GUI) based on Java script (Supplementary Data 1). The GUI renders SIM image in series of the selected ROI with mouse scrolling (①). The contrast of each channel can be adjusted by the setting minimum and maximum pixel values (②). The color map, pixel values, and normalized pixel values of the selected line (vertical white line in the image panel) are visualized on the right-hand column of the GUI (③). Each SIM image series of ROI was loaded with the GUI for users’ visual determination if the structure is a synapse (True), not a synapse (False) or ambiguous to determine (Fail) by clicking an appropriate button (④). The three-dimensional coordinate of the true synapse is saved.

Supplementary Data 1 Code availability. All data supporting the findings of this study are provided in the main text. All the code is available at https://github.com/GTAEKIM/SIM-PRIUS.
