# Supplementary figures and images for: Efficient and Accurate Synapse Detection With Selective Structured Illumination Microscopy on the Putative Regions of Interest of Ultrathin Serial Sections
Source: Front Neuroanat. 2021 Nov 15;15:759816. doi: 10.3389/fnana.2021.759816 (PMC8634652; doi:10.3389/fnana.2021.759816)

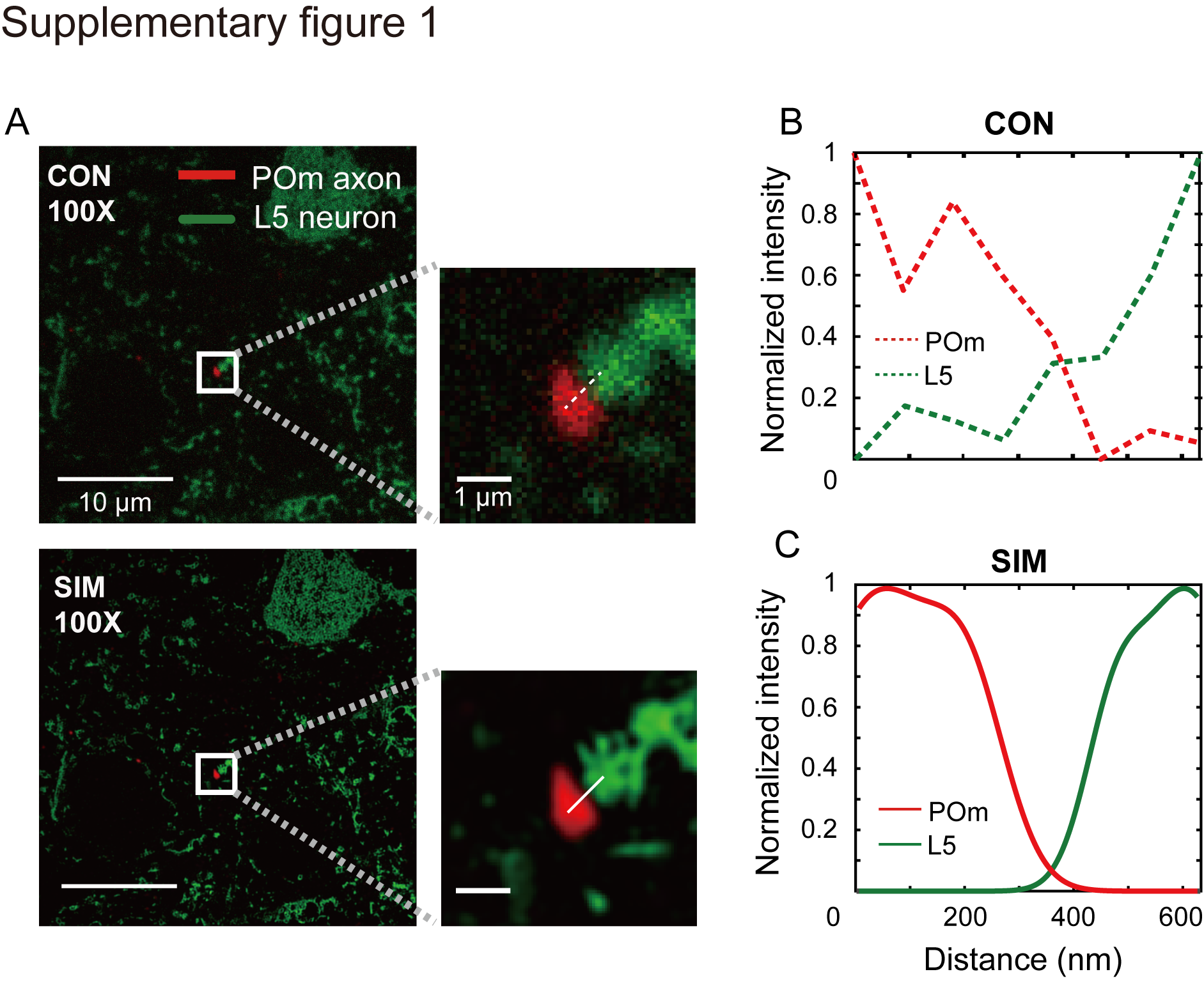

Supplement: Supplementary Figure 1 — Spatial resolution of SIM images. (A) A relatively small pre- and postsynaptic structure image acquired by conventional microscopy (upper) and SIM (lower), and a zoomed-in view (white box, inset). Scale bars represent 10 μm (left) and 1 μm (inset). (B,C) Pixel intensity plots in the magnified images of conventional (dotted) and SIM (solid) images. [file Image_1.TIF]

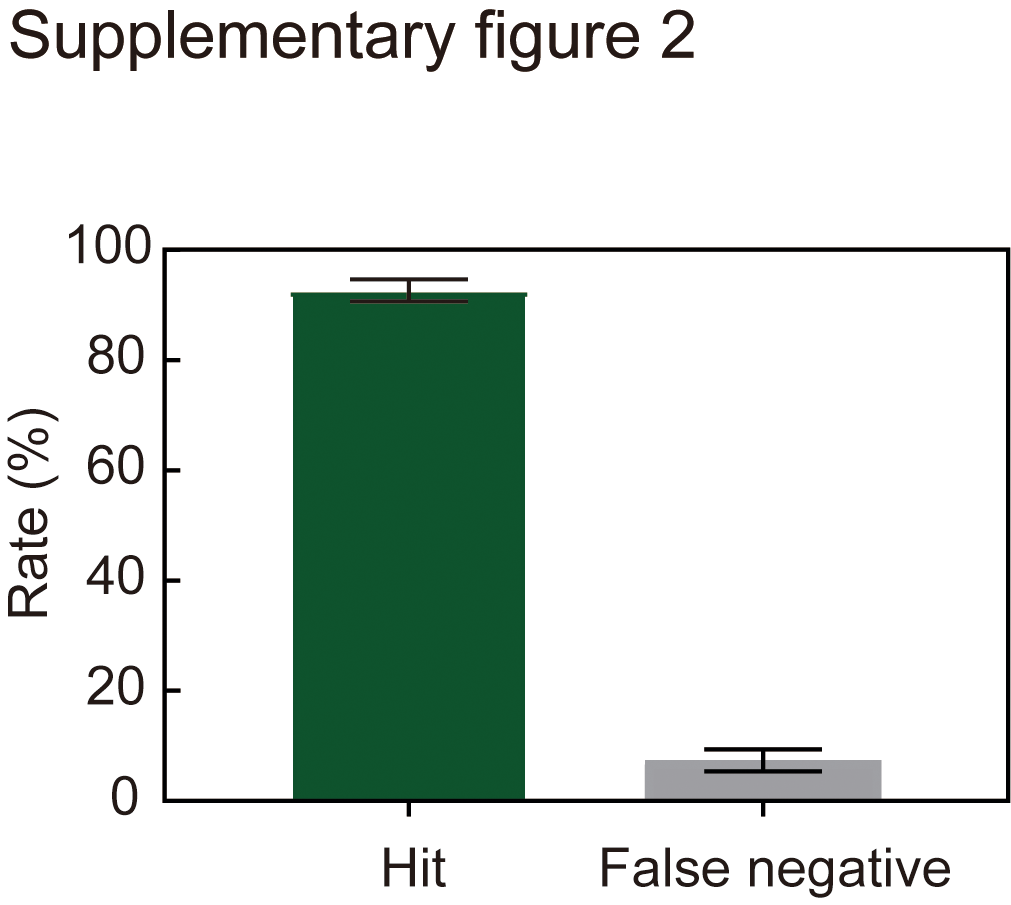

Supplement: Supplementary Figure 2 — Hit and false-negative synapse detection rate of SIM-PRIUS. Comparing of 20x conventional image and 100x SIM images on the same area and quantifying how well the physical connections observed with SIM can be detected in 20x images. [file Image_2.TIF]

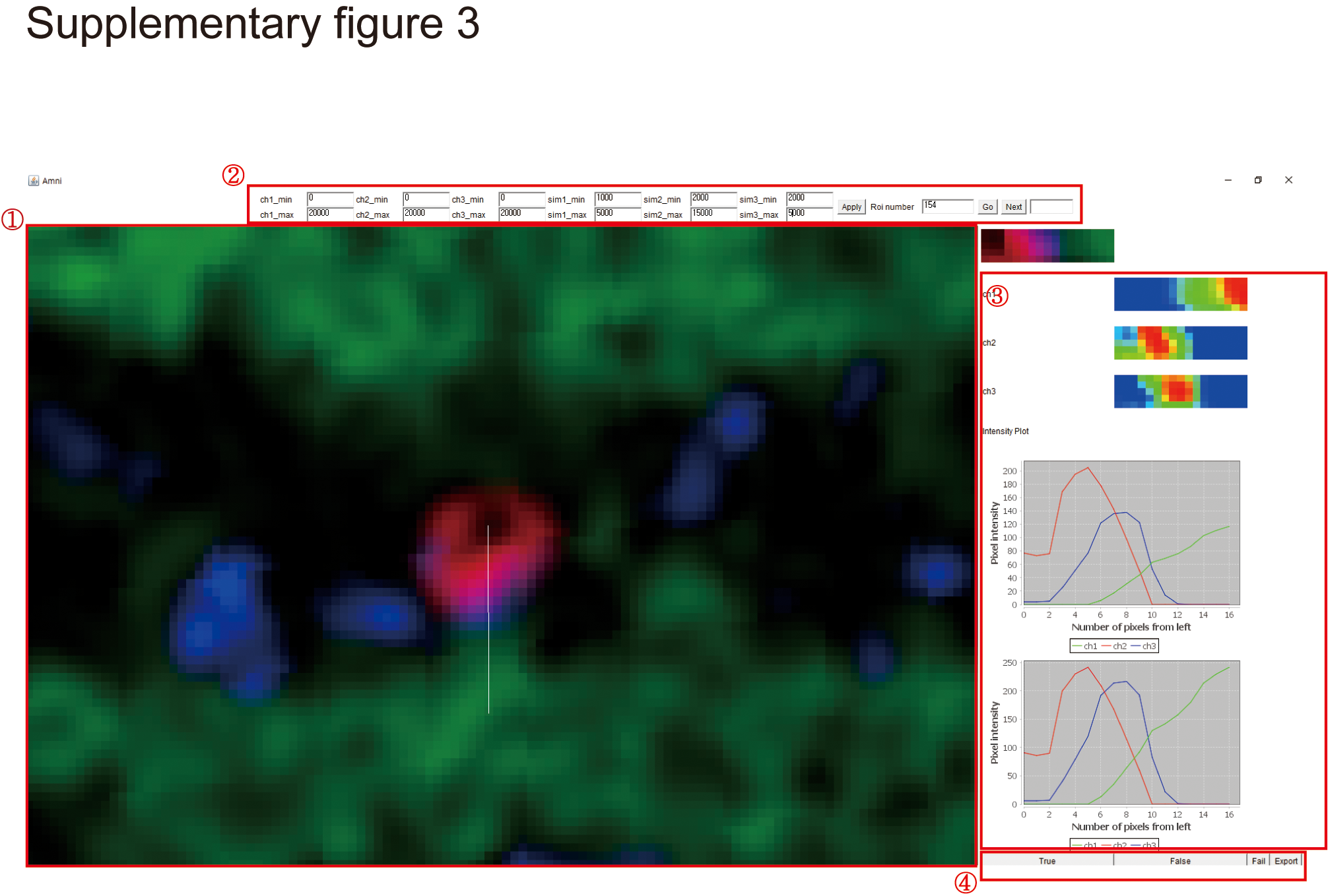

Supplement: Supplementary Figure 3 — Graphical user interface (GUI) for synapse validation. SIM image of the ROI can be visualized with custom-built graphical user interface (GUI) based on Java script (Supplementary Data 1). The GUI renders SIM image in series of the selected ROI with mouse scrolling (①). The contrast of each channel can be adjusted by the setting minimum and maximum pixel values (②). The color map, pixel values, and normalized pixel values of the selected line (vertical white line in the image panel) are visualized on the right-hand column of the GUI (③). Each SIM image series of ROI was loaded with the GUI for users’ visual determination if the structure is a synapse (True), not a synapse (False) or ambiguous to determine (Fail) by clicking an appropriate button (④). The three-dimensional coordinate of the true synapse is saved. [file Image_3.TIF]
